# PGC TagSNP and Its Interaction with *H. pylori* and Relation with Gene Expression in Susceptibility to Gastric Carcinogenesis

**DOI:** 10.1371/journal.pone.0115955

**Published:** 2014-12-31

**Authors:** Cai-yun He, Li-ping Sun, Qian Xu, Jing-wei Liu, Jing-yi Jiang, Nan-nan Dong, Yuan Yuan

**Affiliations:** 1 Tumor Etiology and Screening Department of Cancer Institute and General Surgery, the First Affiliated Hospital of China Medical University, and Key Laboratory of Cancer Etiology and Prevention (China Medical University), Liaoning Provincial Education Department, Shenyang, China; 2 Department of Molecular Diagnostics of Sun Yat-sen University Cancer Center, State Key Laboratory of Oncology in South China, Guangzhou, China; Jawaharlal Nehru University, India

## Abstract

**Background:**

Pepsinogen C (PGC) plays an important role in sustaining the cellular differentiation during the process of gastric carcinogenesis. This study aimed to assess the role of PGC tagSNPs and their interactions with Helicobacter pylori (*H. pylori*) in the development of gastric cancer and its precursor, atrophic gastritis.

**Methods:**

Four PGC tagSNPs (rs6941539, rs6912200, rs3789210 and rs6939861) were genotyped by Sequenom MassARRAY platform in a total of 2311 subjects consisting of 642 gastric cancer, 774 atrophic gastritis, and 895 healthy control subjects. The mRNA and protein expression levels of PGC in gastric tissues and in serum were respectively measured by quantitative reverse transcriptase–polymerase chain reaction (qRT-PCR), immunohistochemistry, and Eenzyme-linked immunoabsorbent assay (ELISA).

**Results:**

We found associations between PGC rs3789210 CG/GG genotypes and reduced gastric cancer risk and between PGC rs6939861 A variant allele and increased risks of both gastric cancer and atrophic gastritis. As for the haplotypes of PGC rs6941539-rs6912200-rs3789210-rs6939861 loci, the TTCA and TTGG haplotypes were respectively associated with increased and reduced risks of both gastric cancer and atrophic gastritis; additionally, the CTCA haplotype was associated with increased atrophic gastritis risk. Very interestingly, rs6912200 CT/TT genotypes had a positive interaction with *H. pylori*, synergistically elevating the gastric cancer risk. Moreover, healthy subjects who carried rs6912200 CT, TT and CT/TT variant genotypes had lower histological and serum expression levels of PGC protein.

**Conclusions:**

Our findings highlight an important role of PGC rs3789210 and rs6939861 in altering susceptibility to atrophic gastritis and/or gastric cancer. Moreover, people who carry rs6912200 variant genotypes exhibit higher gastric cancer risk in case of getting *H. pylori* infection, which strongly suggest a necessity of preventing and/or eliminating *H. pylori* infection in those individuals.

## Introduction

Assessment of both the independent effect of critical gene variations and their joint effect with environmental factors is of great significance to reveal the architecture of gastric cancer predisposition and improve personalized prevention for individuals at risk [1]. Pepsinogen C (PGC or PGII), one of the most important members of aspartic proteinase family, is the precursor of pepsin C that functions as a key digestive enzyme in stomach [2]. Human PGC starts to appear in stomach since the late phase of embryonic development, indicating its involvement in the terminal differentiation of gastric mucosa [3]. The PGC expression in stomach plays an important role in sustaining normal morphology and physiological function of epithelial cells [4]. Our research group previously found that histological PGC protein expression gradually declined with the cellular malignant transformation from the originally normal state to inflammation, precancerous conditions and finally to carcinoma. Additionally, several previous studies in extragastric tissues reported that low-expression of PGC protein was closely related to poor differentiation and unflavored survival in patients with breast, prostate, ovarian, or pancreatic cancers [5]–[8]. These evidences exhibited that PGC has a general role in suppressing the tumor development.

PGC gene is located at chromosome 6p21.3–21.1, encompassing 9 exons and 8 introns (http://www.ncbi.nlm.nih.gov/gene/). So far, twelve common single nucleotide polymorphisms (SNP) have been identified within PGC gene and its extended 5000 bp upstream and downstream sequences by the HapMap project in Chinese Han Beijing population (Release 27,Phase I+II+III, S1 Fig.). Among these twelve SNPs, eight tagging SNPs (tagSNPs) were picked up to efficiently assess the role of PGC polymorphisms in gastric carcinogenesis (S1 Table). We previously identified the associations of three tagSNPs (rs4711690, rs9471643 and rs6458238 polymorphisms) in PGC gene with risk of atrophic gastritis or gastric cancer [9]. However, the roles of the other five tagSNPs (rs6941539, rs6912200, rs3789210, rs6939861 and rs2040017) of PGC gene in susceptibility to gastric cancer remain unknown, which requires further clarification.

Apart from genetic factors, environmental factors are also considered very important in the initiation and progression of gastric cancer, among which H. pylori is thought to be the strongest risk [10]. Moreover, PGC protein has been well-known as a good indicator and effector for *H. pylori* infection and *H. pylori*-related gastropathologies [11]–[13]. For instance, the expression level of serum PGC protein promptly increased when the individual was infected by *H. pylori* while gradually declined and recovered to the baseline level once this microbe was eradicated [11]. Hence, in addition to the PGC genetic variations, the interaction of PGC tagSNP with *H. pylori* infection is also a key component of gastric cancer susceptibility that should not be overlooked.

To comprehensively investigate the role of PGC genetic polymorphisms in altering the susceptibility to gastric cancer, we examined the individual effect of PGC tagSNP and its interaction with *H. pylori* infection on the risks of gastric cancer and its precursor, atrophic gastritis; and then preliminarily explored the influence of PGC tagSNP on its gene expression at both transcriptional and translational levels.

## Materials and Methods

### Sample collection

This study was approved by the human ethics review committee of China Medical University. Written informed consent was obtained from each participant. The subjects enrolled in this research came from the same study population as in our previous study [9]. A full description of the inclusion criteria, diagnosis criteria and characteristics of the study population has been previously described [9]. Briefly, all the included subjects were Chinese residing in northern China, and were recruited from a health check program for gastric cancer screening or from hospitals in Zhuanghe and Shenyang of Liaoning Province, China, between 2002 and 2011. The healthy subjects in the present study comprised individuals with normal stomaches and subjects with only slight or moderate superficial gastritis without atrophic or intestinal metaplasia lesions. Subjects who had a history of other malignancies were excluded. For the genetic association study, a total of 2311 subjects consisting of 642 gastric cancer, 774 atrophic gastritis and 895 healthy control subjects were included. For the PGC mRNA study, 38 patients with gastric cancer were enrolled for the analysis of *PGC* mRNA, from whom cancerous gastric tissues and corresponding noncancerous tissues that were at least 5 cm away from the tumor edge were collected under gastrectomy at the First Affiliated Hospital of China Medical University between 2009 and 2011. For the histological PGC protein study, 226 healthy subjects were randomly selected from the health check program for gastric cancer screening in Zhuanghe performed in 2002, 2008 and 2009. These healthy subjects were endoscopically and histologically identified to have normal mucosa or only slight superficial gastritis without evidence of gastrointestinal symptoms. Biopsy specimens were obtained under gastroscopy from gastric body, angulus and antrum of each subject for the detection of PGC protein *in situ*. For the serum PGC protein study, all the study samples with available serum were included, and finally a total of 1850 subjects consisting of 832 healthy subjects, 737 atrophic gastritis and 281 gastric cancer cases were analyzed.

### Genotyping of *PGC* tagSNPs

Genomic DNA was isolated from peripheral blood lymphocytes by the routine phenol–chloroform method as previously described [9]. Each DNA sample was diluted to a working concentration of 50 ng/µl for genotyping. Assay design and SNP genotyping were performed by Biomiao (Beijing, China) using the Sequenom MassARRAY platform (Sequenom, San Diego, CA, USA) according to the manufacturer's instructions. All samples were randomized on 384-well plates and blinded for disease status. Fifty randomly selected samples were repeatedly genotyped, and the results were 100% concordant.

### Quantitative detection of *PGC* mRNA

The detailed method for the measurement of *PGC* mRNA is described in our previous study [9]. In brief, total RNA from approximately 50 mg cancerous or noncancerous specimens was isolated using TRIzol reagent (Life Technologies, Carlsbad, CA, USA). About 1.5 µg total RNA was converted into complementary DNA using a Quantscript RT kit (Tiangen Biotech, Beijing, China). The mRNA levels for *PGC* and an internal-control gene, glyceraldehyde 3-phosphate dehydrogenase (*GAPDH*), were examined using SYBR Premix Ex Taq II (TaKaRa Biotech, Dalian, China) in an Eppendorf Mastercycler Gradient System (Eppendorf AG, Hamburg, Germany) according to the manufacturer's protocol. Melting curve analysis was performed to exclude the presence of nonspecific products and primer-dimers. No-template controls were included in each experiment. The relative quantification of mRNA levels was calculated using the 2^−ΔΔCt^ method [14].

### Immunohistochemistry (IHC) and semi-quantitative assessment of PGC protein *in situ*

For the retrieval of antigens, 5 µm thick sections were cut from formalin-fixed, paraffin-embedded tissue samples. Sections were dewaxed by heating in citrate buffer (pH 6.0) for 10 min using a microwave. Overnight incubation at 4°C was carried out for the binding of primary antibody (PGII, anti-pepsinogen C antibody, trade name: 2D5, 1∶400 dilution; this antibody was donated by the Japan Clinical Inspection Institute) [15], [16]. Streptavidin-peroxidase two-step immunostaining was then performed according to kit instructions (Kit-9801D2, Maixin Company, Fujian, China). A more detailed method for detecting PGC protein *in situ* has been described previously [17].

Image-Pro Plus software (version 6.0, Media Cybernetics, Silver Spring, MD, USA) was used to quantify the IHC staining of PGC protein. The level of PGC protein was calculated based on the product of the average staining intensity from various images, minimizing possible variation in the staining detection. Using identical microscope and camera settings, four digital images of different fields (400× objective lens) from each sample were taken to accurately reflect the overall staining. To count the exact area of specific PGC staining in each image, the discrimination plane was set at 0–100 in the H channel, 0–255 in the S channel, and 0–200 in I channel. Other nonspecific areas were masked using the setting of 255, 0, 0. To measure the optical density, the original color image was converted to a gray scale image (S2 Fig.). The integrated optical density (IOD) of all the positive PGC staining in each gray scale image and the area of interest (AOI) of PGC staining was measured. The mean density (IOD/AOI) represented the concentration of specific PGC protein in each image. The mean density of four digital images from different fields was finally calculated to represent the average value of PGC protein in each sample.

### ELISA assessment of *H. pylori*-Immunoglobin (Ig) G and PGC protein in serum

Serum *H. pylori*-IgG and PGC protein concentrations were determined as described previously [13] by ELISA (Eenzyme-linked immunoabsorbent assay) (*H. pylori*–IgG ELISA kit and PGII ELISA kit, BIOHIT, Helsinki, Finland) respectively. A reading >34 enzyme immune-units was assumed to be *H. pylori* seropositive.

### Statistical analysis

The Hardy-Weinberg Equilibrium (HWE) of the genotype distribution of each SNP was detected by chi-square test in the control group. The odds ratio (OR) and corresponding 95% interval confidence (CI) were calculated to measure the strength of association between genotype and the risks of gastric cancer and atrophic gastritis. The genetic effect of a single tagSNP on disease risk was detected by multivariate regression analysis controlling for the sex, age and status of *H. pylori* infection. For the haplotype analysis of the four PGC tagSNPs (rs6941539-rs6912200-rs3789210-rs6939861), we set the other haplotypes pooled together as a reference, and assessed the genetic effect of each haplotype with a frequency of at least more than 0.03 in healthy controls, which also controlled for the sex, age and status of *H. pylori* infection. And likelihood ratio tests were performed to assess the interaction effect between genotype and *H. pylori* by comparing the model that only involved the main effects with the full model that also contained the interaction term. To evaluate the association between genotype and gene expression, the distribution of each variable was first tested by the Kolmogorov-Smirnov test. Accordingly, for PGC mRNA data and PGC protein in serum which deviated from a normal distribution, their medians were compared between two groups using Mann-Whitney U test; while for the PGC protein data which fitted a normal distribution, their means were compared using Student's t test. Additionally, the correlation between genotypes and PGC protein in serum were evaluated using partial correlation controlling for sex, age and status of *H. pylori* infection.

All the analyses mentioned above were performed using SPSS 13.0 software (SPSS, Chicago, IL, USA) except for that the haplotype analysis was performed under UNPHASED version 3.1.5 software [18]. All P values were two sided, and P values <0.05 were considered statistically significant.

## Results

### Individual effect of a single PGC tagSNP on the risks of gastric cancer and atrophic gastritis

Because of the confliction of primers in multiplex PCR, rs2040017 polymorphism could not be detected according to the original design, and four PGC tagSNPs, rs6941539, rs6912200, rs3789210 and rs6939861, were successfully genotyped and analyzed in this study. We first assessed the individual analysis of each polymorphism (Table 1). As a result, the AG, AA and AG/AA genotypes of rs6939861 were associated with increased risks of both gastric cancer and atrophic gastritis compared with the common GG genotype (all P<0.05). In addition, rs3789210 CG/GG genotypes were marginally associated with reduced risk of atrophic gastritis compared with the common CC genotype (P = 0.048). We did not observe statistical association between rs6912200 and rs6914539 genotypes and disease risk (all P>0.05).

**Table 1:** Association between a single PGC tagSNP and risks of atrophic gastritis and gastric cancer.

PGC tagSNP	CON(%)	GA(%)	GC(%)	GA vs. CON		GC vs. CON	
				OR(95%CI)	P	OR(95%CI)	P
rs3789210							
CC	674(75.3)	612(79.1)	487(75.9)	1		1	
CG	202(22.6)	148(19.1)	143(22.3)	0.80(0.62,1.03)	0.084	1.00(0.77,1.30)	0.983
GG	19(2.1)	14(1.8)	12(1.9)	0.63(0.31,1.31)	0.217	0.64(0.29,1.40)	0.262
CG/GG				**0.78(0.61,1.00)**	**0.048**	0.96(0.74,1.24)	0.750
rs6912200							
CC	227(25.4)	198(25.7)	166(26.0)	1		1	
CT	448(50.2)	385(50.0)	318(49.8)	1.06(0.82,1.35)	0.673	0.92(0.70,1.20)	0.519
TT	217(24.3)	187(24.3)	155(24.3)	1.07(0.80,1.44)	0.661	0.96(0.71,1.30)	0.796
CT/TT				1.06(0.84,1.34)	0.637	0.93(0.72,1.19)	0.547
rs6939861							
GG	390(45.8)	298(39.7)	246(39.6)	1		1	
AG	396(46.5)	373(49.7)	313(50.4)	**1.25(1.01,1.56)**	**0.045**	**1.29(1.02,1.62)**	**0.034**
AA	65(7.6)	79(10.5)	62(10.0)	**1.62(1.11,2.37)**	**0.013**	**1.53(1.01,2.31)**	**0.044**
AG/AA				**1.30(1.06,1.61)**	**0.014**	**1.32(1.05,1.65)**	**0.015**
rs6941539							
CC	641(72.1)	531(69.1)	460(71.8)	1		1	
CT	227(25.5)	221(28.7)	164(25.6)	1.17(0.93,1.47)	0.187	1.04(0.81,1.34)	0.739
TT	21(2.4)	17(2.2)	17(2.7)	0.96(0.48,1.91)	0.911	1.17(0.59,2.35)	0.655
CT/TT				1.15(0.92,1.44)	0.221	1.05(0.83,1.35)	0.672

All tests were adjusted by age, sex and status of *H. pylori* infection. The results highlighted in bold show associations with disease risk (P values <0.05). Abbreviation: CON: healthy controls; GA: atrophic gastritis, GC: gastric cancer.

### Joint effect of multiple PGC tagSNPs on the risks of gastric cancer and atrophic gastritis

Next, we assessed the effect of common haplotypes of PGC rs6941539-rs6912200-rs3789210-rs6939861 loci on disease risk. Seven haplotypes with frequencies of at least more than 0.03 in control group were observed and analyzed (Table 2). With the other haplotypes pooled together as a reference, we found that both TTCA and CTCA haplotypes were associated with an increased atrophic gastritis risk (P = 0.001 and 0.041, respectively) while TTGG haplotype was related with a reduced atrophic gastritis risk (P = 0.023). For gastric cancer, the TTCA haplotype was associated with an increased gastric cancer risk (P = 0.021) and the TTGG haplotype was associated with a reduced gastric cancer risk (P = 0.017).

**Table 2:** Association between haplotypes of PGC tagSNPs and risks of atrophic gastritis and gastric cancer.

Haplotype^b^	CON	GA	GC	GA vs. CON		GC vs. CON	
				OR(95%CI)^a^	P^a^	OR(95%CI)	P^a^
TTCA	70(4.18%)	100(6.76%)	74(6.00%)	**1.78(1.20,2.65)**	**0.001**	**1.70(1.11,2.60)**	**0.021**
TTCG	467(27.84%)	364(24.62%)	326(26.63%)	0.86(0.72,1.03)	0.112	0.96(0.80,1.16)	0.670
TTGG	65(3.90%)	45(3.05%)	27(2.19%)	**0.59(0.37,0.94)**	**0.023**	**0.54(0.31,0.94)**	**0.017**
TCCA	166(9.88%)	155(10.47%)	126(10.27%)	1.32(1.00,1.73)	0.053	1.17(0.87,1.57)	0.277
TCCG	516(30.73%)	465(31.39%)	366(29.87%)	1.02(0.86,1.21)	0.851	0.92(0.75,1.10)	0.422
TCGA	134(8.01%)	106(7.17%)	101(8.23%)	0.83(0.62,1.12)	0.246	1.00(0.74,1.35)	0.896
CTCA	133(7.90%)	155(10.47%)	111(9.06%)	**1.33(1.01,1.74)**	**0.041**	1.19(0.88,1.61)	0.837
CTCG	105(6.24%)	74(5.03%)	71(5.78%)	0.78(0.54,1.12)	0.178	0.90(0.62,1.30)	0.216

a , P, OR and corresponding 95%CI for individual haplotype test compares with the remaining haplotype pooled together;

b , haplotype of Haplotype of rs6941539 C>T-rs6912200 C>T-rs3789210 C>G-rs6939861 G>A. All tests were adjusted by age, sex and status of H. pylori infection. The results highlighted in bold show associations with disease risk (P values <0.05). Abbreviation: CON: healthy controls; GA: atrophic gastritis, GC: gastric cancer.

### Interaction effect between PGC tagSNP and *H. pylori* on the risks of gastric cancer and atrophic gastritis

We further performed both stratification analysis and interaction analysis to investigate whether the genetic effect of PGC tagSNP on disease risk was modified by the status of *H. pylori* infection. In the stratified analysis according to *H. pylori* infection, rs3789210 CG and CG/GG genotypes showed statistical associations with a reduced atrophic gastritis risk in the subjects without *H. pylori* infection (P = 0.020 and 0.021 respectively); and rs6939861 AA and AG/AA genotypes were associated with an increased risk of gastric cancer (P = 0.042 and 0.034 respectively)(Table 3). We next used Breslow-Day test to compare the difference in the odds ratios between *H. pylori* negative and positive subgroups. Consequently, rs6912200 CT, TT and CT/TT genotypes exhibited statistically different effects on gastric cancer risk between the two subgroups (P = 0.038, 0.025 and 0.017, respectively), suggesting that *H. pylori* had a modifying effect on rs6912200 genotypes (Table 3). Subsequently, interaction analysis confirmed that rs6912200 CT/TT genotypes had a positive interaction with *H. pylori* infection with an interacted OR of 1.75 (P = 0.030), suggesting a synergistic risk effect of rs6912200 CT/TT genotypes and *H. pylori* infection on gastric cancer development (Table 4). The individuals with CT/TT genotype and *H. pylori* infection demonstrated a substantially increased risk of gastric cancer compared with the subjects with CC genotype but without *H. pylori* infection (OR = 2.48, 95%CI:1.77,3.48) (Table 4).

**Table 3:** Stratification analysis of PGC tagSNP with atrophic gastritis and gastric cancer risks according to the status of *H. pylori* infection.

PGC tagSNP	No. of Con/GA/GC		GA vs. CON					GC vs. CON				
	*H. pylori* (−)	*H. pylori* (+)	*H. pylori* (−)		*H. pylori* (+)		P^b^	*H. pylori* (−)		*H. pylori* (+)		P^b^
			OR(95CI%)^a^	*P* a	OR(95CI%)^a^	*P* a		OR(95CI%)^a^	*P* a	OR(95CI%^)a^	*P* a	
rs3789210												
CC	481/253/236	193/359/251	1		1			1		1		
CG	149/52/71	53/96/72	**0.66(0.46,0.94)**	**0.020**	1.03(0.70,1.51)	0.899	0.144	0.97(0.69,1.35)	0.850	1.04(0.68,1.59)	0.856	0.782
GG	9/4/6	10/10/6	0.82(0.25,2.71)	0.750	0.58(0.23,1.42)	0.231	0.550	1.12(0.38,3.36)	0.834	0.39(0.13,1.16)	0.089	0.144
CG/GG			0.67(0.48,0.94)	**0.021**	0.95(0.66,1.37)	0.781	0.242	0.98(0.71,1.35)	0.885	0.93(0.62,1.39)	0.722	0.866
rs6912200												
CC	154/72/91	73/126/74	1		1			1		1		
CT	319/164/146	129/221/172	1.10(0.79,1.54)	0.582	1.01(0.70,1.46)	0.958	0.684	0.76(0.54,1.06)	0.106	1.23(0.81,1.87)	0.336	**0.038**
TT	164/71/74	53/116/81	0.92(0.62,1.370	0.680	1.31(0.84,2.05)	0.229	0.294	0.73(0.49,1.070	0.104	1.56(0.94,2.58)	0.084	**0.025**
CT/TT			1.04(0.75,1.430	0.825	1.09(0.78,1.54)	0.611	0.899	0.74(0.54,1.02)	0.066	1.31(0.88,1.95)	0.186	**0.017**
rs6939861												
GG	274/116/113	116/182/133	1		1			1		1		
AG	286/150/162	110/223/151	1.23(0.92,1.66)	0.163	1.29(0.93,1.79)	0.134	0.851	1.32(0.98,1.78)	0.073	1.24(0.85,1.79)	0.264	0.555
AA	46/32/32	19/47/30	1.64(1.00,2.71)	0.052	1.58(0.88,2.84)	0.128	0.916	1.72(1.02,2.90)	**0.042**	1.28(0.66,2.49)	0.457	0.620
AG/AA			1.29(0.97,1.71)	0.078	1.33(0.97,1.820)	0.082	0.890	1.37(1.02,1.84)	**0.034**	1.24(0.87,1.77)	0.238	0.513
rs6941539												
CC	459/210/221	182/321/239	1		1			1		1		
CT	160/90/83	67/131/81	1.23(0.90,1.670	0.190	1.08(0.76,1.530	0.675	0.660	1.13(0.82,1.56)	0.465	0.92(0.62,1.38)	0.697	0.527
TT	16/6/9	5/11/8	0.78(0.30,2.02)	0.604	1.25(0.43,3.69)	0.683	0.565	1.12(0.47,2.64)	0.798	1.28(0.39,4.23)	0.690	0.953
CT/TT			1.19(0.88,1.60)	0.263	1.09(0.77,1.53)	0.623	0.780	1.13(0.83,1.54)	0.452	0.95(0.64,1.40)	0.782	0.554

a , tests were adjusted by sex and age;

b , P value for BreslowDay test comparing the difference of OR of *H. pylori* negative subgroup with OR of *H. pylori* positive subgroup. The results highlighted in bold show associations with disease risk (P values <0.05). Abbreviation: CON: healthy controls; GA: atrophic gastritis, GC: gastric cancer.

**Table 4:** Interaction effect of PGC tagSNP and *H. pylori* on risks of atrophic gastritis and gastric cancer.

PGC tagSNP		GA vs. CON		GC vs. CON	
		*H.pylori*(−)	*H. pylori*(+)	*H. pylori*(−)	*H. pylori*(+)
rs3789210					
CG/GG	No. of controls/cases	158/56	63/106	158/77	63/78
	OR(95%CI)	1(ref)	5.10(3.28,7.93)	1(ref)	2.83(1.80,4.43)
CC	No. of controls/cases	481/253	193/359	481/236	193/251
	OR(95%CI)	1.50(1.07,2.12)	5.44(3.82,7.76)	1.02(0.74,1.42)	3.02(2.13,4.28)
		P for interaction = 0.177, OR = 0.71		P for interaction = 0.871, OR = 1.04	
rs6912200					
CC	No. of controls/cases	154/72	73/126	154/92	73/74
	OR(95%CI)	1(ref)	3.75(2.50,5.62)	1(ref)	1.91(1.24,2.95)
CT/TT	No. of controls/cases	483/235	182/337	483/220	182/253
	OR(95%CI)	1.03(0.75,1.430	4.08(2.92,5.71)	0.74(0.54,1.02)	2.48(1.77,3.48)
		P for interaction = 0.826, OR = 1.05		**P for interaction = 0.030, OR = 1.75**	
rs6939861					
GG	No. of controls/cases	274/116	116/182	274/113	116/133
	OR(95%CI)	1(ref)	3.82(2.77,5.26)	1(ref)	3.00(2.12,4.25)
AG/AA	No. of controls/cases	332/182	129/270	332/194	129/181
	OR(95%CI)	1.28(0.97,1.70)	5.08(3.75,6.90)	1.38(1.03,1.85)	3.73(2.68,5.18)
		P for interaction = 0.854, OR = 1.04		P for interaction = 0.660, OR = 0.90	
rs6941539					
CC	No. of controls/cases	459/210	182/321	459/221	182/239
	OR(95%CI)	1(ref)	4.00(3.12,5.12)	1(ref)	3.06(2.35,3.99)
CT/TT	No. of controls/cases	176/96	72/142	176/92	72/89
	OR(95%CI)	1.19(0.88,1.60)	4.41(3.18,6.14)	1.13(0.83,1.54)	2.91(2.01,4.21)
		P for interaction = 0.735, OR = 0.93		P for interaction = 0.496, OR = 0.84	

The Interaction effect were assessed by the likelihood ratio test, comparing the fit of the logistic model that included the main effects of sex, age, *H. pylori* and PGC genotype with a fully parameterized model containing the multiplicative interaction terms of genotype and *H. pylori*. The results highlighted in bold show associations with disease risk (P values <0.05). Abbreviation: CON: healthy controls; GA: atrophic gastritis, GC: gastric cancer.

### Correlation between PGC tagSNP and PGC expression at both mRMA and protein levels

We preliminarily explored the influence of PGC tagSNP on gene expression at mRNA and protein levels. We first examined PGC mRNA in 38 pairs of cancerous tissues and matched non-cancerous tissues at least 5 cm apart from the edge of tumorous lesions. However, we did not observe any statistical correlation between the four PGC tagSNPs and PGC mRNA expression level in these samples (all P>0.05, table 5).

**Table 5:** Correlation between PGC tagSNP and levels of PGC mRNA expression.

	N	Non-cancerous tissue			N	Cancerous tissue		
PGC tagSNP		Median(25%, 75%)				Median(25%, 75%)		
		ΔCt	2^−ΔΔCt^	P		ΔCt (Mean ± SD)	2^−ΔΔCt^	P
rs3789210								
CC	33	0.44(−1.50, 2.12)	0.74(0.23, 2.90)		33	0.81(−0.40, 3.78)	0.57(0.07, 1.32)	
CG	5	−1.11(−1.85, 4.74)	2.16(0.42, 3.69)	0.675	5	2.62(0.32, 7.52)	0.16(0.07, 0.98)	0.353
rs6912200								
CC	9	0.65(−0.94, 2.96)	0.64(0.15, 1.92)		9	0.10(−0.86, 5.01)	0.93(0.03, 1.85)	
CT	21	−0.66(−2.14, 3.45)	1.58(0.09, 4.52)	0.563	21	1.04(−0.45, 3.33)	0.49(0.10, 1.37)	0.965
TT	8	0.35(−1.61, 1.46)	0.79(0.37, 3.56)	0.541	8	1.93(0.31, 3.67)	0.29(0.08, 0.81)	0.673
CT/TT	29	0.00(−1.98, 2.06)	1.00(0.24, 3.97)	0.499	29	1.14(−0.25, 3.33)	0.45(0.10, 1.03)	0.840
rs6939861								
GG	19	0.65(−2.46, 3.59)	0.64(0.08, 5.50)		19	0.52(−1.18, 3.54)	0.70(0.09, 2.27)	
AG	15	−0.66(−1.57, 1.56)	1.58(0.34, 2.97)	0.451	15	2.62(0.10, 4.34)	0.16(0.05, 0.93)	0.190
AA	4	1.10(−0.52, 3.36)	0.52(0.12, 1.53)	0.667	4	0.71(0.49, 1.88)	0.62(0.30, 0.71)	0.725
AG/AA	19	−0.24(−1.11, 1.75)	1.18(0.30, 2.16)	0.644	19	1.26(0.45, 4.01)	0.42(0.06, 0.73)	0.212
rs6941539								
CC	24	0.13(−2.06, 1.70)	0.92(0.31, 4.19)		24	1.25(−0.53, 3.58)	0.42(0.08, 1.46)	
CT	13	0.65(−1.37, 3.70)	0.64(0.08, 2.62)	0.337	13	0.69(−0.40, 3.53)	0.62(0.22, 1.32)	0.561
TT	1	−0.98	1.97	0.720	1	5.68	0.20	0.240
CT/TT	14	0.65(−1.28, 3.64)	0.64(0.08, 2.44)	0.427	14	0.75(−0.33, 5.71)	0.60(0.02, 1.26)	0.800

All tests were used Mann-Whitney U test.

We further investigated the influence of genotypes on PGC protein levels. Serum PGC protein levels were detected by ELISA in a total of 1850 subjects consisting of 832 controls, 737 atrophic gastritis and 281 gastric cancer patients (table 6). Considering normal mucosa and/or mild superficial gastritis as an ascertained benign condition that was minimally affected by other confounding factors, 226 healthy subjects offering a total of 284 specimens were analyzed for histological PGC protein. To further eliminate the confounding effect of particular locations on gastric tissues, we divided the tissue specimens into three subgroups according to their locations: 75 gastric body, 72 gastric angulus and 137 gastric antrum specimens (table 7). The overall analysis of PGC protein found no statistical correlation between PGC tagSNP and PGC protein expression either in the 284 tissue specimens detected by immunohistochemistry or in the 1850 serum samples detected by ELISA. In the subgroup analysis of histological PGC protein in immunohistochemistry experiment, we observed that the subjects who carried rs6912200 CT, TT and CT/TT genotypes had lower histological levels of PGC protein in gastric body specimens (P = 0.014, 0.032 and 0.042, respectively). Similarly, in the subgroup analysis of serum PGC protein in ELISA experiment, we found correlations between PGC rs6912200 variant genotype and decreased expression levels of PGC protein in controls subjects (P = 0.026). Moreover, PGC rs6941539 CT and CT/TT genotypes had decreased expression levels of PGC protein in gastric cancer patients (P = 0.007 and 0.014, respectively).

**Table 6:** Correlation between PGC tagSNP and levels of PGC protein expression in serum.

SNP	Total				Healthy subject				Atrophic gastritis				Gastric cancer			
	N	Median(25%,75%)	P^a^	P^b^	N	Median(25%,75%)	P^a^	P^b^	N	Median(25%,75%)	P^a^	P^b^	N	Median(25%,75%)	P^a^	P^b^
rs3789210				0.883				0.853				0.507				0.627
CC	1421	9.30(6.00,16.50)			629	7.70(5.30,11.88)			584	11.70(6.87,19.30)			208	12.65(7.01,21.84)		
CG	391	9.20(6.00,17.00)	0.871		185	7.49(5.10,10.15)	0.364		141	12.40(6.97,20.50)	0.564		65	16.00(8.35,24.29)	0.119	
GG	38	9.70(6.55,17.00)	0.741		18	8.85(5.95,16.48)	0.212		12	12.10(6.20,17.05)	0.439		8	11.98(7.05,23.15)	0.890	
CG/GG	429	9.20(6.07,17.00)	0.727		203	7.50(5.10,10.50)	0.596		153	12.40(6.80,19.40)	0.723		73	14.98(8.17,24.29)	0.159	
rs6912200				0.300				**0.026**				0.603				0.582
CC	470	10.05(6.10,16.72)			218	8.10(5.38,12.37)			188	12.45(7.51,19.90)			64	10.28(7.33,23.90)		
CT	931	9.20(5.80,16.70)	0.177		416	7.68(5.30,11.70)	0.467		368	11.40(6.20,18.88)	**0.039**		147	14.50(7.04,23.27)	0.271	
TT	449	9.00(6.20,16.35)	0.248		198	7.25(5.09,9.77)	0.055		181	11.80(7.75,19.85)	0.512		70	13.50(7.23,22.07)	0.420	
CT/TT	1380	9.10(5.90,16.68)	0.153		614	7.50(5.20,10.73)	0.196		549	11.60(6.60,19.20)	0.083		217	14.20(7.07,22.95)	0.271	
rs6939861				0.296				0.278				0.268				0.829
GG	795	9.00(5.90,16.90)			384	7.50(5.32,11.70)			297	12.80(6.95,18.50)			114	14.30(6.38,23.07)		
AG	888	9.30(6.03,16.28)	0.979		383	7.70(5.20,11.70)	0.938		361	11.00(6.60,18.50)	0.167		144	13.33(7.63,23.58)	0.784	
AA	167	10.46(6.70,16.30)	0.334		65	8.07(5.60,11.30)	0.839		79	13.06(8.10,19.30)	0.736		23	12.30(7.04,23.30)	0.829	
AG/AA	1055	9.40(6.10,16.30)	0.800		448	7.80(5.20,11.68)	0.990		440	11.45(6.70,18.50)	0.280		167	13.20(7.60,23.30)	0.851	
rs6941539				0.302				0.369				0.786				0.304
CC	1327	9.30(6.20,16.74)			604	7.70(5.40,11.60)			514	11.85(6.90,19.30)			209	14.40(7.75,23.55)		
CT	481	9.20(5.70,15.89)	0.147		208	7.88(5.20,11.88)	0.828		207	11.40(6.50,18.36)	0.541		66	9.12(5.41,19.18)	**0.007**	
TT	42	10.35(5.58,18.68)	0.929		20	6.00(4.95,10.45)	0.253		16	18.35(8.47,23.64)	0.243		6	17.80(8.95,29.50)	0.687	
CT/TT	523	9.20(5.70,15.90)	0.163		228	7.79(5.19,11.70)	0.618		223	11.70(6.70,19.40)	0.751		72	10.10(5.53,21.67)	**0.014**	

a , P values for Mann-Whitney Test comparing the difference of variables between two groups;

b , P values for partial correlation analysis controlling for sex, age and status of H. pylori infection. The results highlighted in bold show associations with expression levels of PGC protein in serum (P values <0.05).

**Table 7:** Correlation between PGC tagSNP and levels of PGC protein expression in situ.

PGC tagSNP	Total (N = 284)			Gastric corpus (N = 75)			Gastric angulus (N = 72)			Gastric antrum (N = 137)		
	N	Mean ± SD	P	N	Mean ± SD	P	N	Mean ± SD	P	N	Mean ± SD	P
rs3789210												
CC	228	0.194±0.048		58	0.226±0.046		63	0.192±0.044		107	0.177±0.044	
CG	47	0.192±0.053	0.841	14	0.244±0.055	0.196	7	0.172±0.021	0.225	26	0.169±0.037	0.420
GG	9	0.210±0.050	0.320	3	0.265±0.041	0.149	2	0.181±0.024	0.704	4	0.183±0.029	0.771
CG/GG	56	0.195±0.053	0.860	17	0.248±0.052	0.093	9	0.017±0.021	0.214	30	0.171±0.036	0.517
rs6912200												
CC	68	0.198±0.158		17	0.260±0.067		18	0.188±0.033		33	0.172±0.036	
CT	147	0.194±0.048	0.614	41	0.224±0.038	**0.014**	37	0.190±0.045	0.825	69	0.178±0.047	0.492
TT	69	0.189±0.042	0.291	17	0.217±0.036	**0.032**	17	0.019±0.045	0.757	35	0.173±0.036	0.847
CT/TT	216	0.192±0.046	0.422	58	0.222±0.037	**0.042**	54	0.191±0.045	0.785	104	0.177±0.044	0.566
rs6939861												
GG	122	0.192±0.048		30	0.227±0.045		34	0.189±0.048		58	0.174±0.040	
AG	136	0.198±0.052	0.271	41	0.234±0.052	0.536	31	0.190±0.038	0.930	64	0.179±0.047	0.536
AA	26	0.180±0.033	0.258	4	0.221±0.015	0.784	7	0.193±0.034	0.868	15	0.164±0.024	0.327
AG/AA	162	0.195±0.050	0.498	45	0.233±0.050	0.589	38	0.191±0.037	0.892	79	0.176±0.044	0.788
rs6941539												
CC	185	0.194±0.047		48	0.228±0.039		49	0.185±0.037		88	0.180±0.046	
CT	94	0.195±0.054	0.887	27	0.235±0.061	0.542	22	0.201±0.051	0.151	45	0.168±0.033	0.109
TT	5	0.163±0.021	0.144	NA	NA	NA	1	0.195	0.785	4	0.155±0.013	0.284
CT/TT	99	0.193±0.054	0.908	NA	NA	NA	23	0.201±0.050	0.148	49	0.167±0.032	0.071

All tests used Student-T test. The results highlighted in bold show associations with expression levels of PGC protein in situ (P values <0.05).

## Discussion

This study highlights an important role of PGC genetic variations in the susceptibility to gastric cancer. We newly found associations between PGC rs3789210 CG/GG genotypes and reduced risk of gastric cancer and between PGC rs6939861 A variant allele and increased risks of both atrophic gastritis and gastric cancer. As for the haplotypes of PGC rs6941539-rs6912200-rs3789210-rs6939861 loci, TTCA and TTGG haplotypes were respectively associated with increased and reduced risks of both gastric cancer and atrophic gastritis and CTCA haplotype was associated with an increased risk of atrophic gastritis. Very interestingly, rs6912200 CT/TT genotypes had a positive interaction with *H. pylori* infection, synergistically elevated the risk of gastric cancer although this polymorphism had no main effect on disease risk. Moreover, healthy subjects who carried rs6912200 variant genotypes had lower histological and serum levels of PGC protein.

It is currently known that there are at least two different types of polymorphisms for PGC gene, including insertion/deletion and SNP. In 1993, Azuma et al initially reported that a 100 bp insertion/deletion within intron 7 of PGC gene was associated with an increased risk of gastric ulcer [19]. Subsequently, both Liu et al's and Sun et al's studies independently found its deletion allele was linked with an increased gastric cancer risk and lower histological levels of PGC protein [20]–[22]. As for the PGC SNP, in addition to PGC rs4711690, rs6458238 and rs9471643 identified previously [9], our study newly found rs3789210 in intron 3 and rs6939861 in 3′ downstream of PGC gene had main effects on susceptibility to gastric cancer. So far, the rs6939861 polymorphism was the only SNP that was found to be associated with increased risks of gastric cancer and atrophic gastritis, with ORs ranging from 1.25 to 1.62. It is worth noting that the genotype frequencies distribution was deviated from Hardy-Weinberg equilibrium (HWE) in control subjects. There are two main possible interpretations accounting for this phenomenon, including the inappropriate selection of genotyping method or a lack of representativeness of selected population. Considering the case of the former interpretation, we found that the genotyping by Sequenom MassARRAY in this study showed a concordance of 100% of repeated detection for rs6939861. Additionally, the PCR-based sequencing in 5 random samples had validated the correction of genotyping for this SNP. Such quality control precludes the possibility of uncorrected genotyping of rs6939861 in this study. As for the control subjects in this study, they had two different sources. One part of the control subjects were recruited from the hospitals that underwent physical examination in Shenyang city and the other part of the control subjects were selected randomly from the participants in a health check program for gastric cancer screening in Zhuanghe County. It seems to be one possible interpretation for a deviation of rs6939861 from HWE in this study. Nevertheless, our findings for rs6939861 should be further validated in future study with a larger study sample size.

Apart from the host genetics, environmental factors also play a critical role in the development of gastric cancer. The effect of one confirmed risk environmental factor, *H. pylori* infection, has been concerned in this study. The present study observed that the PGC rs6912200 variant T allele had lower histological levels of PGC protein in gastric body tissues but it still did not exhibit any independent genetic effect on disease risk. Nevertheless, once infected by *H. pylori*, the subject carrying PGC rs6912200 variant genotypes has an increased risk of suffering from gastric cancer. In recent years, PGC protein has been considered as an important effector of *H. pylori* infection in stomach [11], [12], [23]. Ning et al reported that the histological expression levels of PGC protein were prone to be affected by *H. pylori* [16]. They found that histological PGC protein levels increased in acute *H. pylori* infection while declined in chronic *H. pylori* infection in accompany with the reduction of gland cells. *H. pylori* infection seems to play an essential role in the initiation of the genetic activity of PGC rs6912200 polymorphism. Our research group previously found another interaction effect between PGC rs4711690 polymorphism and *H. pylori* infection in the development of atrophic gastritis [9]. Those evidences collectively suggest that PGC genetic variations may function as important effectors that respond to certain virulence factor of *H. pylori* and thereby affect the strength of host response to *H. pylori* infection. Previous studies also suggested that *H. pylori* could directly induce a series of pathophysiologic alterations in gastric epithelium or indirectly change the biological function or expression pattern of host gene via certain virulence factors, both of which advance the development of gastric cancer and its precursor, atrophic gastritis [24], [25]. However, the biological mechanism of interaction between PGC polymorphism and *H. pylori* infection during the process of gastric carcinogenesis is still unclear, which requires further molecular function studies to elucidate. It is worth mentioning that the resistance against *H. pylori* pathogenicity appears to be weakened among population who carry the rs6912200 variant genotypes. We should, therefore, take targeted prevention and elimination of *H. pylori* infection for those individuals seriously.

Influence of polymorphism on the gene expression at mRNA or protein level is one of the most common molecular mechanisms underlying the association between genotype and disease risk. Based on such a hypothesis, we explored to investigate whether the PGC polymorphisms are associated with PGC mRNA and protein expressions. We observed that rs6912200 in the 5′ upstream of PGC gene was related with the PGC protein level by both immunohistochemistry and ELISA detections. However, our results showed that the mRNA or protein results were not exactly accordant with the genetic association of rs3789210 and rs6939861. There are several possible explanations for this discordance. The sample used for expression experiments in this study is relatively small, which may underestimate the influence of polymorphism on gene expression. Also, we are wondering if there is other mechanism underlying this association that still could not be established by the present experiments. The fact that rs3789210 is located in intron region and the rs6939861 is located in 3′ downstream raised the possibility that there is a third factor such as non-coding RNA and splice factor involved in manipulating the function of PGC gene. Usually such factors display their gene modifying functions in specific conditions. So far we could not draw a definite conclusion on the molecular function for the associated polymorphisms.

We are aware that there are several major limitations in this study. First, the genotype-expression associations were not exactly accordant with the genotype-disease associations for the rs3789210 and rs6939861. This difference may be due to the limited sample size for the expression experiments. Also, there may be other mechanisms underlying these associations that still could not yet be established by mRNA or protein experiments. Second, in histological PGC protein experiments, we only enrolled the tissues extracted from healthy subjects other than from the cases with diseased stomaches (here referred to atrophic gastritis and gastric cancer patients). Our major considerations are as follows. Besides the host genetics, histological PGC expression could also be affected by several other factors such as status of gastric mucosa, location of affected or selected tissues, and state of *H. pylori* infection. Especially, the status of gastric mucosa and the location of tissue have significant effects on in situ PGC expression levels as described in previous studies [16], [17]. Therefore, detection of histological PGC protein in cancerous tissues could not be accomplished in experimental practice. By contrast, the normal mucosa/mild superficial gastritis is an ascertained benign condition that has minimal impact by other confounding factors. Therefore, we considered the subjects with endoscopically- and histopathologically-confirmed normal stomach and slight superficial gastritis as eligible samples for PGC expression experiment in situ.

Taking together, this study found for the first time that PGC rs3789210 was associated with a reduced risk of atrophic gastritis and PGC rs6939861 was associated with increased risks of both atrophic gastritis and gastric cancer. There were joint effects of PGC rs6941539-rs6912200-rs3789210-rs6939861 loci, demonstrating genetic roles of TTCA, TTGG and CTCA haplotypes in altering susceptibility to atrophic gastritis and/or gastric cancer. Moreover, PGC rs6912200 CT/TT genotypes had a positive interaction with *H. pylori* infection in conferring an increased risk of gastric cancer, and individuals carrying such genotypes had lower levels of histological and serum PGC protein expression. Our findings indicate a further research direction for PGC genetic variation in gastric carcinogenesis, and also provide an important clue for personalized *H. pylori* management in the overall strategy to reduce the high prevalence of gastric cancer.

## Supporting Information

S1 Fig **The location of the studied 4 SNPs.** All the SNPs within PGC gene and its extended 5000 bp upstream and downstream regions identified in the HapMap project in Chinese Han Beijing population (Release 27,Phase I+II+III). This figure showed a visual appearancemanifestation for the location of the studied 4 SNPs which was a supplement for the Supplementary table 1. The tagSNP rs6939861 located in promoter region of PGC gene, rs3789210 located in intron 6, and rs6941539 and rs6912200 located in 3′ untranslated region of PGC gene.
(TIF)

S2 Fig **Original color image and converted gray image.** Positive expression of PGC protein in the cytoplasm of gastric gland cells (immunohistochemical staining ×400). Original color image was converted to a gray scale image before measuring integrated optical density and area of interest of all the positive PGC staining in each gray scale image. (A and B) Original color image and gray scale image in gastric body; (C and D): Original color image and gray scale image in gastric angulus; and (E and F): Original color image and gray scale image in gastric antrum.
(TIF)

S1 Table **TagSNPs for PGC gene according to genotype data of HapMap project in Chinese Han Beijing population.**
(DOCX)

## References

[1] Roukos DH (2009) Assessing both genetic variation (SNPs/CNVs) and gene-environment interactions may lead to personalized gastric cancer prevention. Expert Rev Mol Diagn 9:1–6. 19099341 10.1586/14737159.9.1.1

[2] Gritti I, Banfi G, Roi GS (2000) Pepsinogens: physiology, pharmacology pathophysiology and exercise. Pharmacol Res 41:265–281. 10675278 10.1006/phrs.1999.0586

[3] Yasugi S (2000) Epithelial cell differentiation during stomach development. Hum Cell 13:177–184. 11329933

[4] Massarrat S, Haj-Sheykholeslami A, Mohamadkhani A, Zendehdel N, Aliasgari A, et al (2014) Pepsinogen II Can Be a Potential Surrogate Marker of Morphological Changes in Corpus before and after H. pylori Eradication. Biomed Res Int 2014:481607. 25028655 10.1155/2014/481607 PMC4083213

[5] Scorilas A, Diamandis EP, Levesque MA, Papanastasiou-Diamandi A, Khosravi MJ, et al (1999) Immunoenzymatically determined pepsinogen C concentration in breast tumor cytosols: an independent favorable prognostic factor in node-positive patients. Clin Cancer Res 5:1778–1785. 10430082

[6] Diaz M, Rodriguez JC, Sanchez J, Sanchez MT, Martin A, et al (2002) Clinical significance of pepsinogen C tumor expression in patients with stage D2 prostate carcinoma. Int J Biol Markers 17:125–129. 12113579 10.1177/172460080201700208

[7] Rojo JV, Merino AM, Gonzalez LO, Vizoso F (2002) Expression and clinical significance of pepsinogen C in epithelial ovarian carcinomas. Eur J Obstet Gynecol Reprod Biol 104:58–63. 12128264 10.1016/s0301-2115(01)00610-8

[8] Truan N, Vizoso F, Fresno MF, Fernandez R, Quintela I, et al (2001) Expression and clinical significance of pepsinogen C in resectable pancreatic cancer. Int J Biol Markers 16:31–36. 11288952 10.1177/172460080101600104

[9] He C, Tu H, Sun L, Xu Q, Li P, et al (2013) Helicobacter pylori-related host gene polymorphisms associated with susceptibility of gastric carcinogenesis: a two-stage case-control study in Chinese. Carcinogenesis 34:1450–1457. 23455381 10.1093/carcin/bgt079

[10] Fock KM, Katelaris P, Sugano K, Ang TL, Hunt R, et al (2009) Second Asia-Pacific Consensus Guidelines for Helicobacter pylori infection. J Gastroenterol Hepatol 24:1587–1600. 19788600 10.1111/j.1440-1746.2009.05982.x

[11] Di Mario F, Moussa AM, Cavallaro LG, Caruana P, Merli R, et al (2004) Clinical usefulness of serum pepsinogen II in the management of Helicobacter pylori infection. Digestion 70:167–172. 15479977 10.1159/000081517

[12] Nurgalieva ZZ, Opekun AR, Graham DY (2006) Problem of distinguishing false-positive tests from acute or transient Helicobacter pylori infections. Helicobacter 11:69–74. 16579835 10.1111/j.1523-5378.2006.00380.x

[13] He CY, Sun LP, Gong YH, Xu Q, Dong NN, et al (2011) Serum pepsinogen II: a neglected but useful biomarker to differentiate between diseased and normal stomachs. J Gastroenterol Hepatol 26:1039–1046. 21303408 10.1111/j.1440-1746.2011.06692.x

[14] Livak KJ, Schmittgen TD (2001) Analysis of relative gene expression data using real-time quantitative PCR and the 2(-Delta Delta C(T)) Method. Methods 25:402–408. 11846609 10.1006/meth.2001.1262

[15] Konishi N, Matsumoto K, Hiasa Y, Kitahori Y, Hayashi I, et al (1995) Tissue and serum pepsinogen I and II in gastric cancer identified using immunohistochemistry and rapid ELISA. J Clin Pathol 48:364–367. 7615858 10.1136/jcp.48.4.364 PMC502557

[16] Ning PF, Liu HJ, Yuan Y (2005) Dynamic expression of pepsinogen C in gastric cancer, precancerous lesions and Helicobacter pylori associated gastric diseases. World J Gastroenterol 11:2545–2548. 15849808 10.3748/wjg.v11.i17.2545 PMC4305740

[17] Li P, He C, Sun L, Dong N, Yuan Y (2013) Pepsinogen I and II expressions in situ and their correlations with serum pesignogen levels in gastric cancer and its precancerous disease. BMC Clin Pathol 13:22. 24004680 10.1186/1472-6890-13-22 PMC3846408

[18] Dudbridge F (2008) Likelihood-based association analysis for nuclear families and unrelated subjects with missing genotype data. Hum Hered 66:87–98. 18382088 10.1159/000119108 PMC2386559

[19] Azuma T, Teramae N, Hayakumo T, Yasuda K, Nakajima M, et al (1993) Pepsinogen C gene polymorphisms associated with gastric body ulcer. Gut 34:450–455. 8098309 10.1136/gut.34.4.450 PMC1374301

[20] Liu HJ, Guo XL, Dong M, Wang L, Yuan Y (2003) Association between pepsinogen C gene polymorphism and genetic predisposition to gastric cancer. World J Gastroenterol 9:50–53. 12508350 10.3748/wjg.v9.i1.50 PMC4728248

[21] Sun LP, Guo XL, Zhang Y, Chen W, Bai XL, et al (2009) Impact of pepsinogen C polymorphism on individual susceptibility to gastric cancer and its precancerous conditions in a Northeast Chinese population. J Cancer Res Clin Oncol 135:1033–1039. 19132389 10.1007/s00432-008-0539-3 PMC12160201

[22] Sun LP, Gong YH, Dong NN, Wang L, Yuan Y (2009) Correlation of pepsinogen C (PGC) gene insertion/deletion polymorphism to PGC protein expression in gastric mucosa and serum. Ai Zheng 28:487–492. 19624876

[23] Di Mario F, Cavallaro LG, Moussa AM, Caruana P, Merli R, et al (2006) Usefulness of serum pepsinogens in Helicobacter pylori chronic gastritis: relationship with inflammation, activity, and density of the bacterium. Dig Dis Sci 51:1791–1795. 17203556 10.1007/s10620-006-9206-1

[24] Wen S, Moss SF (2009) Helicobacter pylori virulence factors in gastric carcinogenesis. Cancer Lett 282:1–8. 19111390 10.1016/j.canlet.2008.11.016 PMC2746929

[25] He C, Chen M, Liu J, Yuan Y (2014) Host genetic factors respond to pathogenic step-specific virulence factors of Helicobacter pylori in gastric carcinogenesis. Mutat Res 759C:14–26. 10.1016/j.mrrev.2013.09.002 24076409

