# Supplementary figures and images for: PGC TagSNP and Its Interaction with H. pylori and Relation with Gene Expression in Susceptibility to Gastric Carcinogenesis
Source: PLoS One. 2014 Dec 31;9(12):e115955. doi: 10.1371/journal.pone.0115955 (PMC4281127; doi:10.1371/journal.pone.0115955)

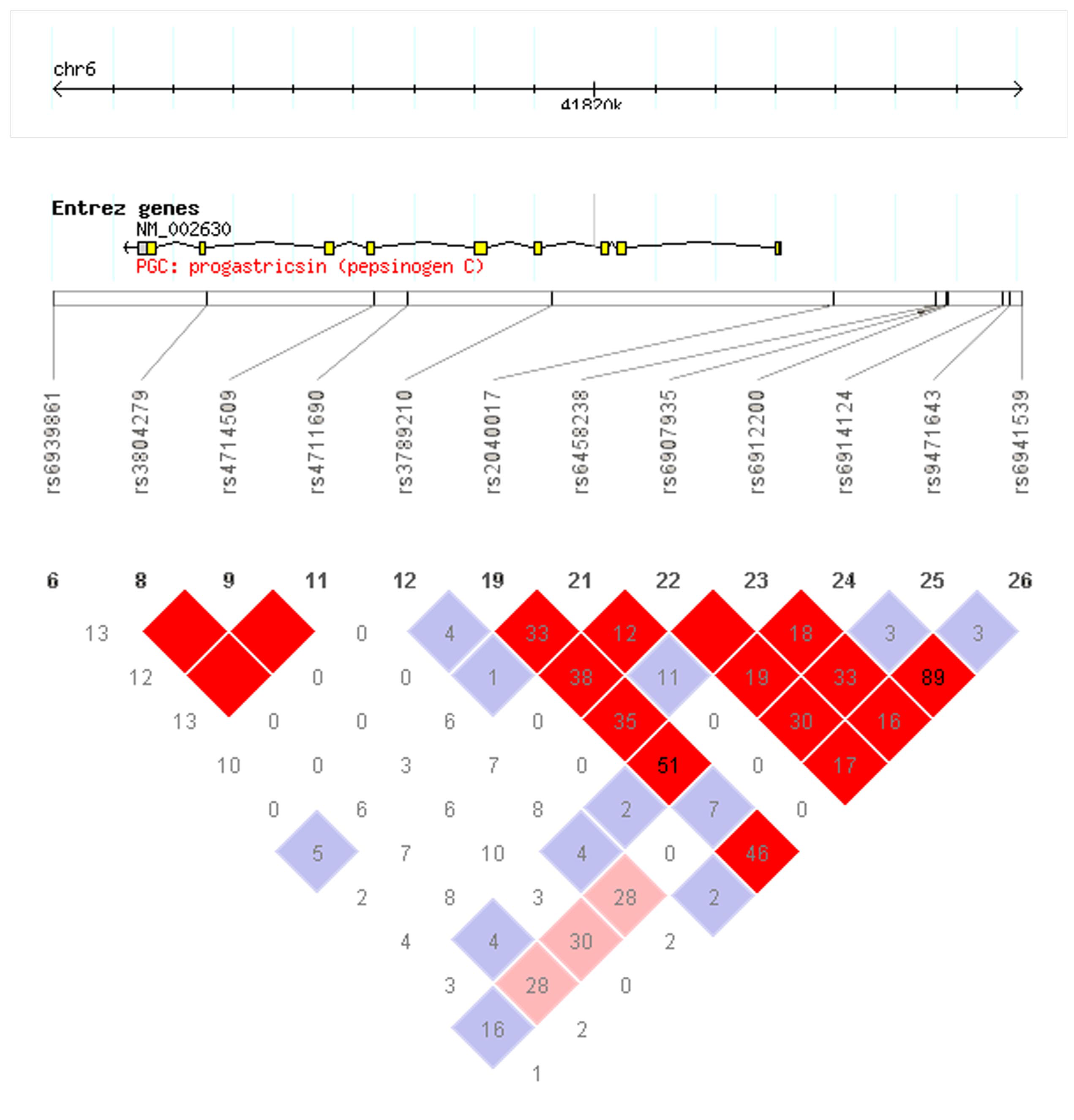

Supplement: S1 Fig — The location of the studied 4 SNPs. All the SNPs within PGC gene and its extended 5000 bp upstream and downstream regions identified in the HapMap project in Chinese Han Beijing population (Release 27,Phase I+II+III). This figure showed a visual appearancemanifestation for the location of the studied 4 SNPs which was a supplement for the Supplementary table 1. The tagSNP rs6939861 located in promoter region of PGC gene, rs3789210 located in intron 6, and rs6941539 and rs6912200 located in 3′ untranslated region of PGC gene. (TIF) [file pone.0115955.s001.tif]

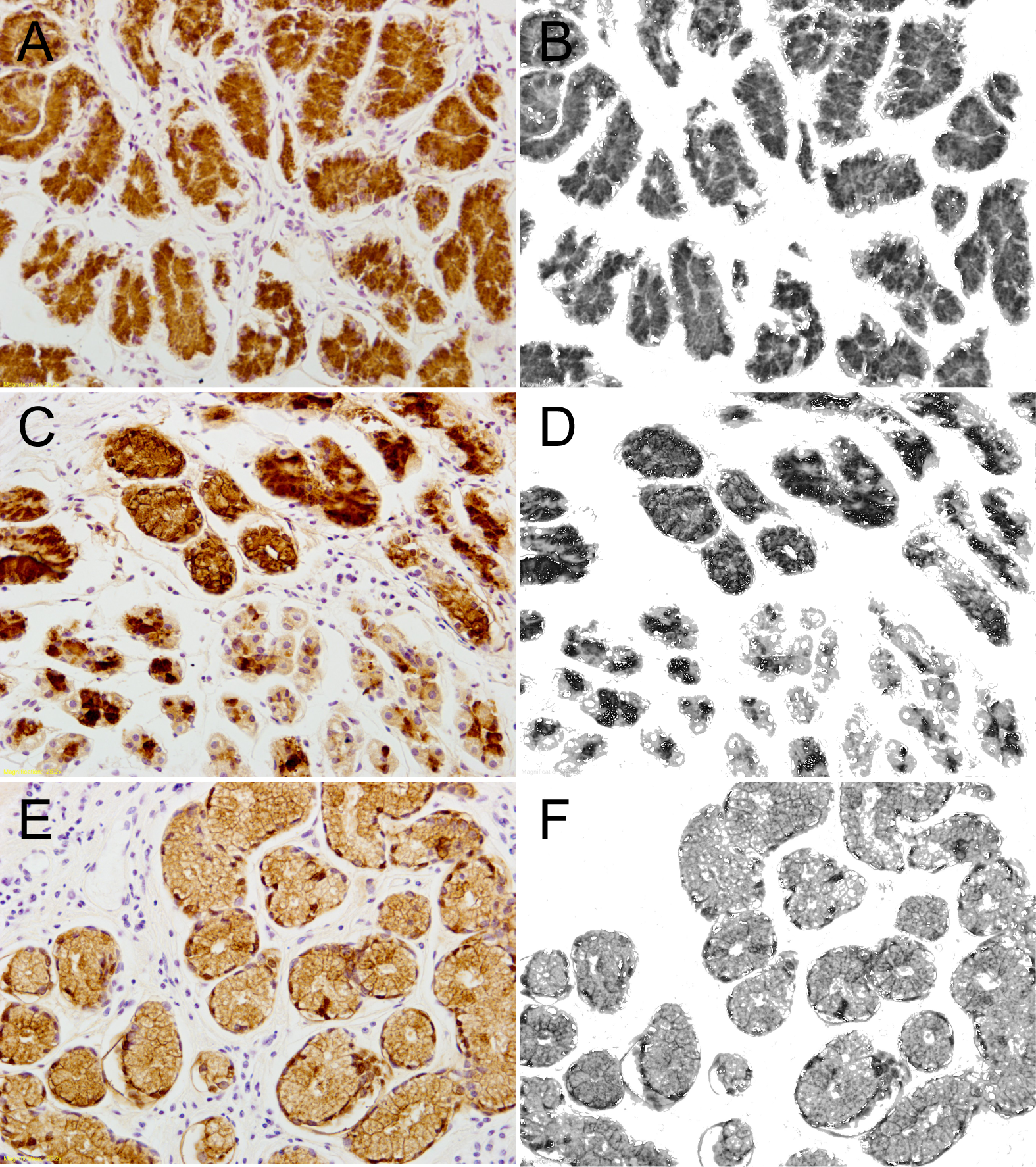

Supplement: S2 Fig — Original color image and converted gray image. Positive expression of PGC protein in the cytoplasm of gastric gland cells (immunohistochemical staining ×400). Original color image was converted to a gray scale image before measuring integrated optical density and area of interest of all the positive PGC staining in each gray scale image. (A and B) Original color image and gray scale image in gastric body; (C and D): Original color image and gray scale image in gastric angulus; and (E and F): Original color image and gray scale image in gastric antrum. (TIF) [file pone.0115955.s002.tif]
