# Supplementary material for: PGC TagSNP and Its Interaction with H. pylori and Relation with Gene Expression in Susceptibility to Gastric Carcinogenesis
Source: PLoS One. 2014 Dec 31;9(12):e115955. doi: 10.1371/journal.pone.0115955 (PMC4281127; doi:10.1371/journal.pone.0115955)
Supplement: S1 Table — TagSNPs for PGC gene according to genotype data of HapMap project in Chinese Han Beijing population. (DOCX) [file pone.0115955.s003.docx]

**Supplementary table 1 TagSNPs for PGC gene according to genotype data of HapMap project in Chinese Han Beijing population**

| TagSNP | Alleles Captured by tagSNP | Position | Prediction function^a^ |
| --- | --- | --- | --- |
| rs4711690 | rs4714509,rs4711690,rs3804279 | Intronic region | Splicing regulation |
| rs6458238 | rs6458238 | 5'upstream | At TF binding site |
| rs9471643 | rs9471643 | 5'upstream | At TF binding site |
| rs6912200 | rs6907935,rs6912200 | 5'upstream | No known function |
| rs3789210 | rs3789210 | Intronic region | No known function |
| rs6939861 | rs6939861 | 5'upstream | No known function |
| rs6941539 | rs6941539,rs6914124 | 5'upstream | No known function |
| rs2040017 | rs2040017 | No known function | No known function |

^a^, the function of each tagSNP was predicted by a bioinformatics software online (FastSNP: http://fastsnp.ibms.sinica.edu.tw/).
